# Cabozantinib prevents AGEs-induced degradation of type 2 collagen and aggrecan in human chondrocytes

**DOI:** 10.18632/aging.205186

**Published:** 2023-12-06

**Authors:** Yang Dong, Lianfang Lin, Yuan Ji, Xu Cheng, Zhiwu Zhang

**Affiliations:** 1 Second Department of Hand Surgery, Yantaishan Hospital, Yantai 264008, Shangdong Province, China

**Keywords:** osteoarthritis, Cabozantinib, extracellular matrix, chondrocytes, SOX-9

## Abstract

Osteoarthritis (OA) is a joint degenerative disease commonly observed in the old population, lacks effective therapeutic methods, and markedly impacts the normal lives of patients. Degradation of extracellular matrix (ECM) is reported to participate in OA development, which is a potential target for treating OA. Cabozantinib is an inhibitor of tyrosine kinases and is recently claimed with suppressive properties against inflammation. Herein, the protective function of Cabozantinib on advanced glycation end products (AGEs)-induced damages to chondrocytes was tested. SW1353 chondrocytes were stimulated with 100 μg/ml AGEs with or without 10 and 20 μM Cabozantinib for 24 h. Signally increased reactive oxygen species (ROS) levels, declined reduced glutathione (GSH) levels, and elevated release of inflammatory cytokines were observed in AGEs-stimulated SW1353 chondrocytes, which were markedly reversed by Cabozantinib. Moreover, the notably reduced type II collagen and aggrecan levels, and increased matrix metalloproteinase-13 (MMP-13) and A Disintegrin and Metalloproteinase with Thrombospondin Motifs-5 (ADAMTS-5) levels in AGEs-stimulated SW1353 chondrocytes were largely rescued by Cabozantinib. The downregulated Sry-type high-mobility-group box 9 (SOX-9) observed in AGEs-stimulated SW1353 chondrocytes was abolished by Cabozantinib. Furthermore, the impact of Cabozantinib on type II collagen and aggrecan levels in AGEs-treated SW1353 chondrocytes was abrogated by silencing SOX-9. Collectively, Cabozantinib prevented AGEs-induced degradation of type 2 collagen and aggrecan in human chondrocytes by mediating SOX-9.

## INTRODUCTION

Osteoarthritis (OA) is an age-related joint degenerative disease characterized by progressive degeneration and destruction of articular cartilage. The main clinical symptoms are joint pain, deformity and dysfunction, and it is the fourth inducer of disability [1]. The overall prevalence of OA is 15%, which increases significantly with age [2]. The pathogenesis of OA is complex and has not been fully elucidated to date. Under the treatment with existing methods, only clinical symptoms of OA are alleviated, with the progressive development of articular cartilage lesions in OA unsolved. In the late stage of severe OA, only joint replacement surgery is effective in remedying the joint dysfunction [3]. Therefore, it is urgent to clarify the pathogenesis and progression mechanism of OA to explore effective therapeutic targets for OA. The prevalence of OA elevates with age, and age is recognized as a prominent risk factor for the initiation and progression of OA [4, 5]. However, the underlying specific mechanism is still unclear. With the increase of age, the production and accumulation of advanced glycation end products (AGEs) is one of the most significant changes in the body, and it is believed that AGEs are the molecular basis of the pathogenesis of OA [6–8]. The biological properties of cartilage tissue are changed by the accumulation of AGEs in articular cartilage tissue, including increased fragility and decreased resistance to shear force and pressure, which is one of the reasons why OA occurs in non-weight-bearing joints or in the normal BMI range [9]. However, AGEs formation and cross-linking inhibitors such as ALT-711 and aminoguanidine exert limited effects in the treatment of OA, suggesting that AGEs might participate in the pathophysiological process of OA through intracellular pathways. Articular cartilage is mainly composed of chondrocytes and ECM, and chondrocytes play a vital role in maintaining the morphology and function of the cartilage. The synthesis and renewal of the cartilage matrix mainly rely on chondrocytes [10]. The synthesis and degradation of normal ECM are always in dynamic equilibrium and ECM is mainly composed of type II collagens and aggrecans. The level of proteolytic enzymes in articular cartilage tissue of OA patients is significantly increased, while the dynamic balance of ECM is broken in OA patients by matrix metalloproteinases (MMPs), which induces the destruction of articular cartilage integrity, and ultimately promotes the development of OA [11]. Regulating the degradation of ECM by type II collagen and aggrecan is beneficial for the treatment of OA.

Cabozantinib is a multi-target small molecule tyrosine kinase inhibitor, including c-MET, VEGFR, ROS1, RET, AXL, etc. [12]. Cabozantinib was approved by the Food and Drug Administration (FDA) for the treatment of advanced thyroid cancer in January 2012, as the second-line treatment in April 2016, and as the first-line treatment in early-stage renal-cell cancer in December 2017. With the advancement of various clinical trials, Cabozantinib plays an important role in the treatment of prostate cancer, liver cancer, and non-small cell lung cancer [13, 14]. Recently, the repressive property of Cabozantinib on inflammation has been reported [15]. Cabozantinib was found to inhibit the activation of the NF-κB pathway and decrease the expression of pro-inflammatory mediators such as TNF-α, IL-6, and MCP-1 [16, 17]. However, the effect of Cabozantinib on OA remains unknown. Our research focuses on the protective function of Cabozantinib on AGEs-induced damages to chondrocytes to investigate the potential role of Cabozantinib in OA treatments.

## RESULTS

### Cytotoxicity of Cabozantinib in SW1353 chondrocytes

**Figure 1:**
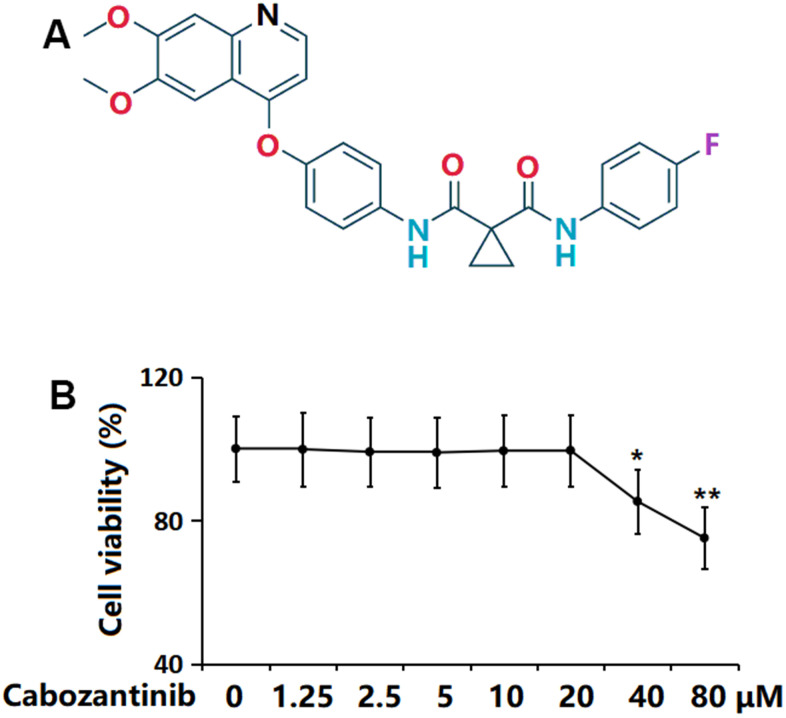
**Cytotoxicity of Cabozantinib in SW1353 chondrocytes.** (**A**) Molecular structure of Cabozantinib; (**B**) Cells were stimulated with Cabozantinib at the concentrations of 0, 1.25, 2.5, 5, 10, 20, 40, 80 μM for 24 hours. The cell viability was measured using the MTT assay (n=6, *, ** *P*<0.05, 0.01 vs. Control group).

The molecular structure of Cabozantinib is visualized in Figure 1A. SW1353 chondrocytes were stimulated with Cabozantinib at the concentrations of 0, 1.25, 2.5, 5, 10, 20, 40, 80 μM for 24 h, followed by detecting the cell viability to determine the optimized concentration. The cell viability of SW1353 chondrocytes was kept around 99% when the concentration was lower than 20 μM but was notably reduced to 85.3% and 75.1% by 40 and 80 μM Cabozantinib, respectively (Figure 1B). Thus, 10 and 20 μM Cabozantinib were applied [18].

### Cabozantinib repressed the oxidative stress (OS) in AGEs-treated SW1353 chondrocytes

**Figure 2:**
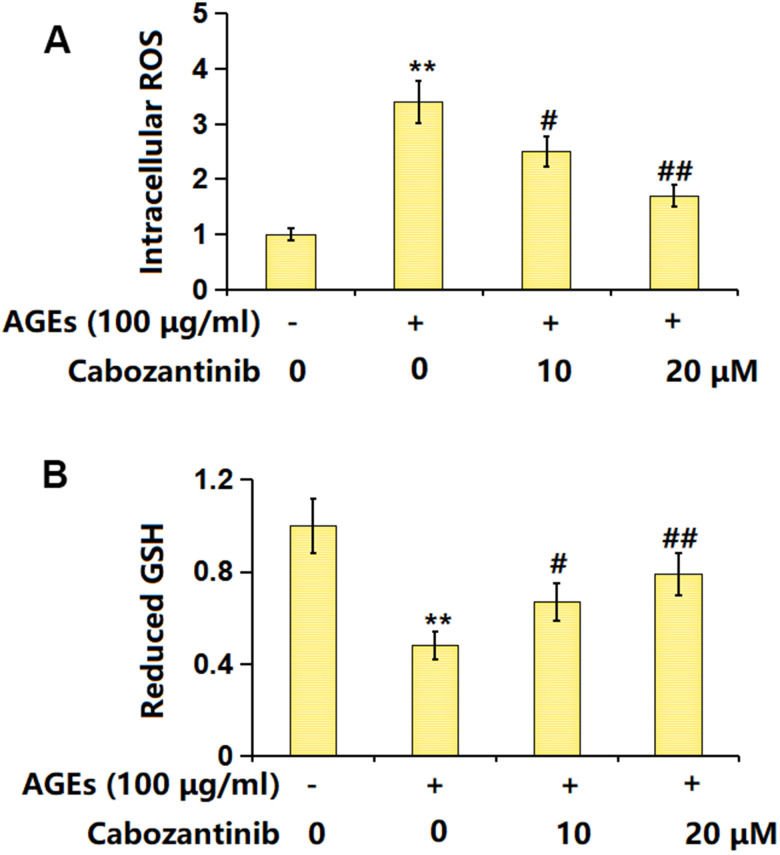
**Cabozantinib repressed the oxidative stress in AGEs-treated SW1353 chondrocytes.** SW1353 chondrocytes were stimulated with 100 μg/ml AGEs with or without 10 and 20 μM Cabozantinib for 24 h. (**A**) Intracellular ROS was measured using DCFH-DA staining; (**B**) The levels of reduced GSH were measured (n=6, *, ** *P*<0.05, 0.01 vs. Control group, #, ## *P*<0.05, 0.01 vs. AGEs group).

SW1353 chondrocytes were stimulated with 100 μg/ml AGEs with or without 10 and 20 μM Cabozantinib for 24 h. The intracellular ROS level was found signally increased in AGEs-stimulated chondrocytes but was markedly reduced by 10 and 20 μM Cabozantinib (Figure 2A). Moreover, the declined level of GSH in AGEs-stimulated chondrocytes was notably increased by 10 and 20 μM Cabozantinib (Figure 2B). A repressive property of Cabozantinib on OS in AGEs-treated SW1353 chondrocytes was observed.

### Cabozantinib inhibited the inflammation in AGEs-treated SW1353 chondrocytes

**Figure 3:**
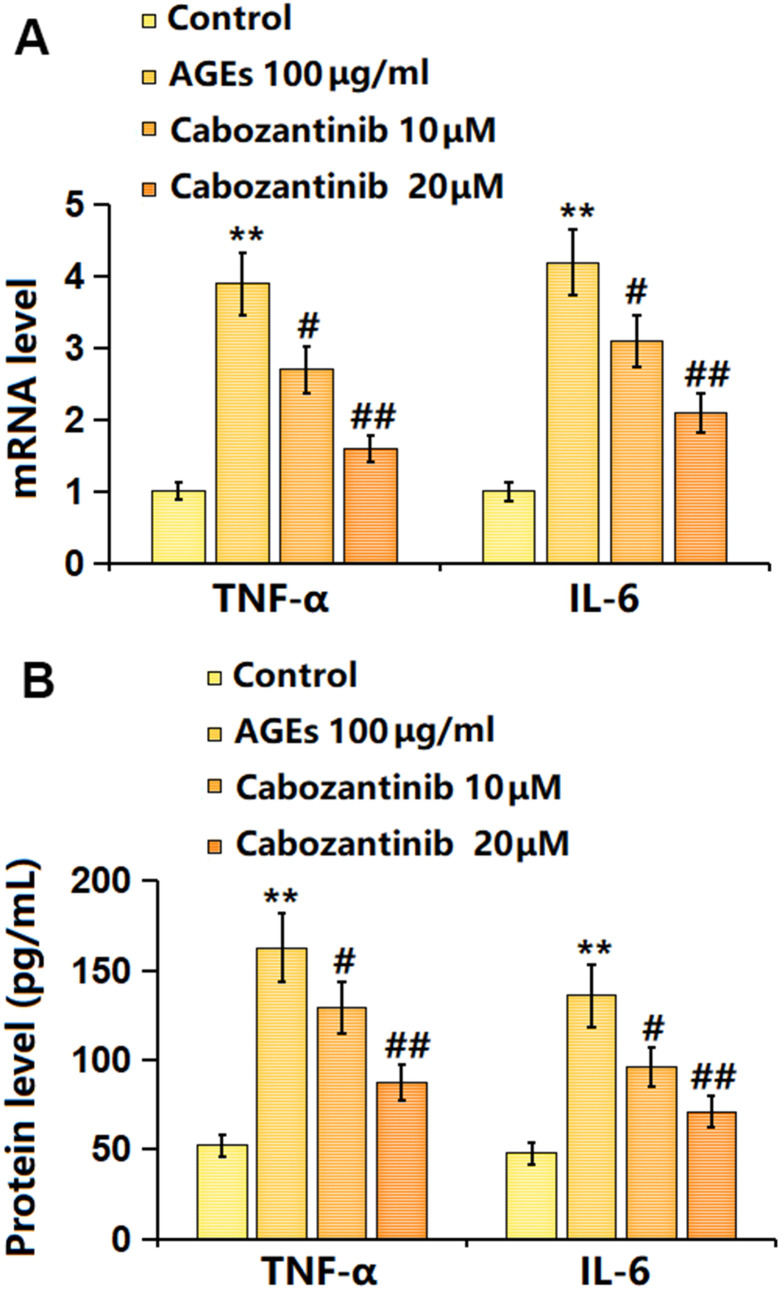
**Cabozantinib inhibited the inflammation in AGEs-treated SW1353 chondrocytes.** SW1353 chondrocytes were stimulated with 100 μg/ml AGEs with or without 10 and 20 μM Cabozantinib for 24 h. (**A**) mRNA level of TNF-α, and mRNA level of IL-6. (**B**) The protein level of TNF-α, and the protein level of IL-6 (n=6, *, ** *P*<0.05, 0.01 vs. Control group, #, ## *P*<0.05, 0.01 vs. AGEs group).

Subsequently, the release of inflammatory cytokines was detected. The mRNA levels of TNF-α and IL-6 were signally increased in AGEs-stimulated SW1353 chondrocytes, then notably decreased by 10 and 20 μM Cabozantinib (Figure 3A). Furthermore, the TNF-α production in AGEs-stimulated SW1353 chondrocytes was elevated from 52.3 to 162.7 pg/mL, which was repressed to 129.4 and 87.4 pg/mL by 10 and 20 μM Cabozantinib, respectively. The IL-6 levels in the control, AGEs, 10 μM Cabozantinib, and 20 μM Cabozantinib groups were 47.8, 135.9, 96.1, and 71.2 pg/mL, respectively (Figure 3B). A suppressive effect of Cabozantinib on inflammation in AGEs-treated SW1353 chondrocytes was observed.

### Cabozantinib increased type II collagen and aggrecan levels in AGEs-treated SW1353 chondrocytes

**Figure 4:**
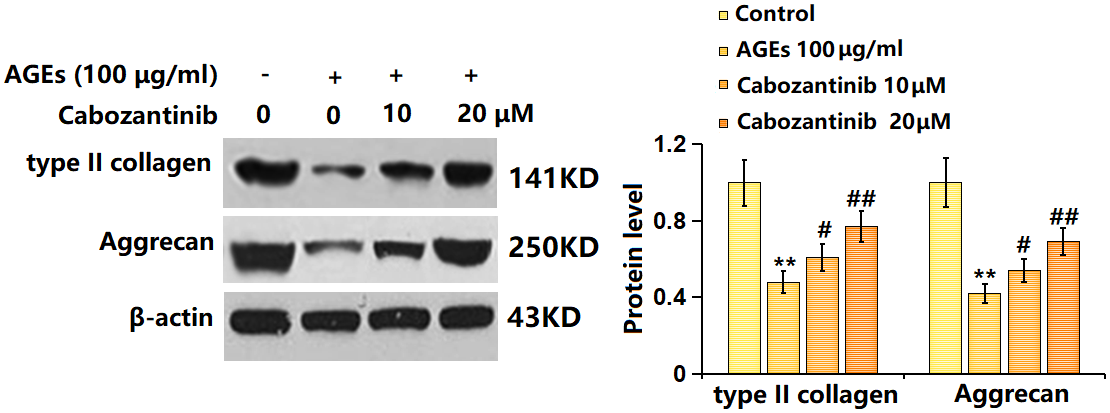
**Cabozantinib increased type II collagen and aggrecan levels in AGEs-treated SW1353 chondrocytes.** SW1353 chondrocytes were stimulated with 100 μg/ml AGEs with or without 10 and 20 μM Cabozantinib for 24 h. The type II collagen and Aggrecan levels were detected by the Western blotting assay (n=6, *, ** *P*<0.05, 0.01 vs. Control group, #, ## *P*<0.05, 0.01 vs. AGEs group).

To check the potential regulatory function of Cabozantinib on OA, the levels of type II collagen and aggrecan were determined. As evidenced by the Western blotting assay, the markedly decreased levels of type II collagen and aggrecan in AGEs-treated SW1353 chondrocytes were largely elevated by 10 and 20 μM Cabozantinib (Figure 4). An elevation effect of Cabozantinib on type II collagen and aggrecan levels in AGEs-treated SW1353 chondrocytes was observed.

### Cabozantinib repressed the levels of MMP-13 and ADAMTS-5 in AGEs-treated SW1353 chondrocytes

**Figure 5:**
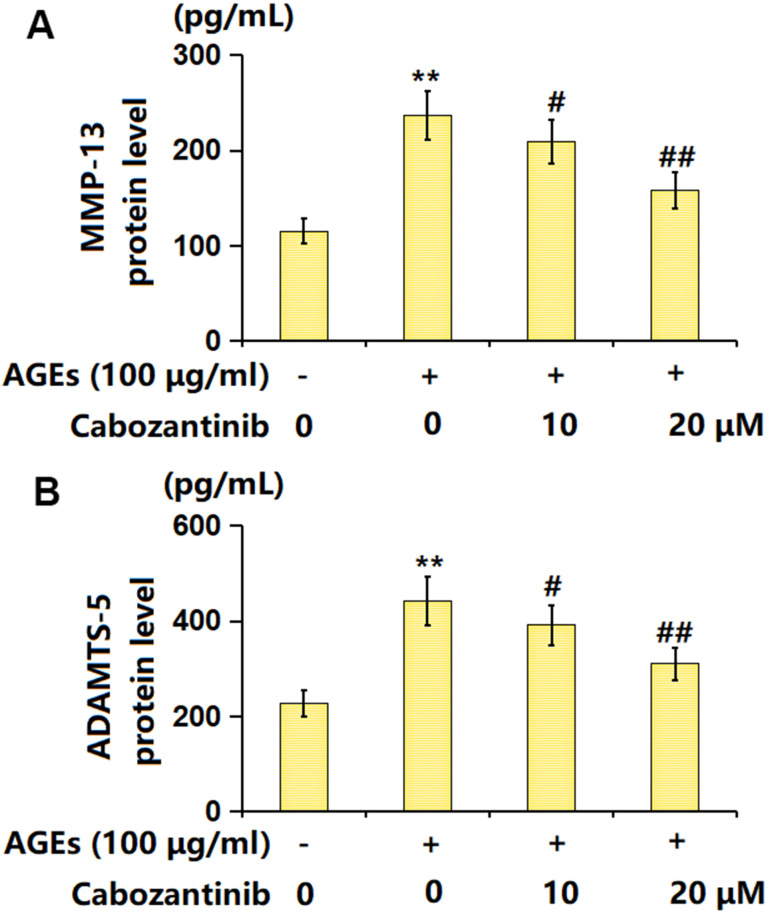
**Cabozantinib repressed the levels of MMP-13 and ADAMTS-5 in AGEs-treated SW1353 chondrocytes.** SW1353 chondrocytes were stimulated with 100 μg/ml AGEs with or without 10 and 20 μM Cabozantinib for 24 h. (**A**) The protein level of MMP-13 was determined by the ELISA assay. (**B**) The protein level of ADAMTS-5 was determined by the ELISA assay (n=6, *, ** *P*<0.05, 0.01 vs. Control group, #, ## *P*<0.05, 0.01 vs. AGEs group).

MMP-13 and ADAMTS-5 are important enzymes for the degradation of ECMs in OA development [19]. The MMP-13 level in AGEs-stimulated chondrocytes was markedly increased from 115.2 to 236.8 pg/mL, which was decreased to 209.5 and 158.3 pg/mL by 10 and 20 μM Cabozantinib, respectively (Figure 5A). Furthermore, the ADAMTS-5 levels in the control, AGEs, 10 μM Cabozantinib, and 20 μM Cabozantinib groups were 226.9, 441.9, 391.5, and 309.6, respectively (Figure 5B). A suppressive effect of Cabozantinib on MMP-13 and ADAMTS-5 levels in AGEs-treated SW1353 chondrocytes was observed.

### Cabozantinib upregulated SOX-9 in AGEs-treated SW1353 chondrocytes

**Figure 6:**
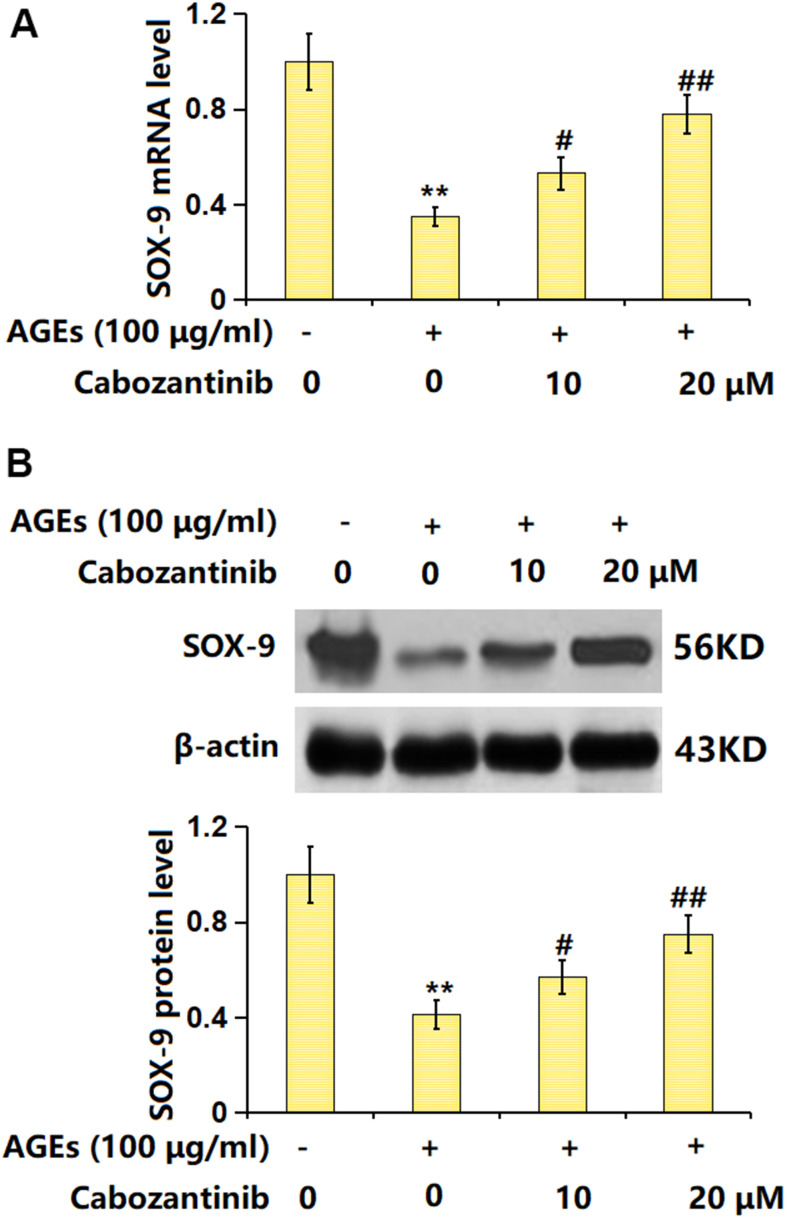
**Cabozantinib upregulated SOX-9 in AGEs-treated SW1353 chondrocytes.** SW1353 chondrocytes were stimulated with 100 μg/ml AGEs with or without 10 and 20 μM Cabozantinib for 24 h. (**A**) mRNA level of SOX-9. (**B**) Protein level of SOX-9 (n=6, *, ** *P*<0.05, 0.01 vs. Control group, #, ## *P*<0.05, 0.01 vs. AGEs group).

SOX-9 is a critical transcription factor responsible for the expression of ECM components [20]. The SOX-9 level was found markedly decreased in AGEs-stimulated chondrocytes but notably increased by 10 and 20 μM Cabozantinib (Figure 6A, 6B), suggesting the function of Cabozantinib in AGEs-treated SW1353 chondrocytes might be correlated with SOX-9.

### Silencing SOX-9 abrogated the function of Cabozantinib on type II collagen and aggrecan levels in AGEs-treated SW1353 chondrocytes

**Figure 7:**
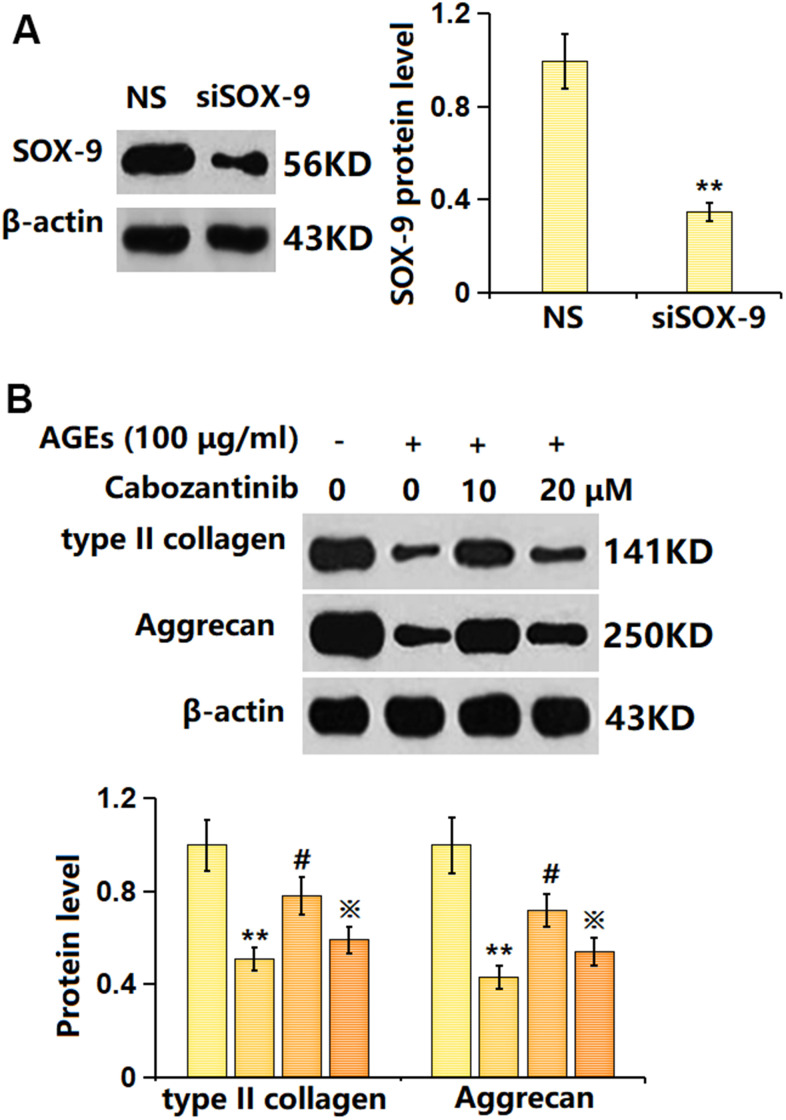
**Silencing SOX-9 abolished the effect of Cabozantinib on type II collagen and aggrecan levels in AGEs-treated SW1353 chondrocytes.** Cells were transfected with the siRNA targeting SOX-9 (siR-SOX-9), followed by stimulated with 100 μg/ml AGEs in the presence or absence of 20 μM Cabozantinib for 24 h. (**A**) Successful knockdown of SOX-9. (**B**) The protein level of type II collagen and Aggrecan was determined by the ELISA assay (n=6, *, ** *P*<0.05, 0.01 vs. Control group, #, ## *P*<0.05, 0.01 vs. AGEs group, ※*P*<0.05 vs. Cabozantinib group).

To verify the involvement of SOX-9, SW1353 chondrocytes were transfected with the siRNA targeting SOX-9 (siR-SOX-9), followed by stimulation with 100 μg/ml AGEs in the presence or absence of 20 μM Cabozantinib for 24 h. The successful knockdown of SOX-9 in chondrocytes was verified as shown in Figure 7A. The type II collagen level in AGEs-stimulated chondrocytes was found decreased to 0.51 in the control group, which was elevated to 0.78 by Cabozantinib. After the silencing of SOX-9, the type II collagen level was markedly reduced to 0.59. Furthermore, the aggrecan levels in the control, AGEs, Cabozantinib, and Cabozantinib+ siR-SOX-9 groups were 1, 0.43, 0.72, and 0.54, respectively (Figure 7B).

## DISCUSSION

A large number of studies have confirmed that the pathogenesis of OA is correlated to ECM degradation, inflammatory factors, and chondrocyte apoptosis [21, 22]. In articular cartilage, chondrocytes are mainly responsible for the synthesis of ECM, so as to ensure the realization of normal joint structure and function [23]. In the development of OA, abnormal expression of inflammatory factors leads to increased apoptosis of chondrocytes and degradation of ECM [24]. The ECM of cartilage is complex, and mainly composed of collagen fibers, water, electrolytes, and proteoglycans [25]. In adult articular cartilages, collagen fibers cross each other to form a network, decomposing the stress on the cartilage. Proteoglycans control the water permeability of cartilage ECM [26]. The surface of proteoglycans is smooth after water absorption, and the external force-induced destruction of cartilage is prevented by such smooth effects [27]. Thus, avoiding the degradation of cartilage ECM contributes to the delay in OA progression [28]. In terms of morphology, cartilage in the juvenile period is usually milky white with a texture, and then gradually changes to yellow, with cracks appearing on its surface. Furthermore, the microstructure of cartilage is altered significantly, accompanied by water loss and degeneration of collagen fibers [29]. With increased OA severity, the synthesis and catabolism of cartilage are seriously unbalanced, coupled with the further increase and accumulation of MMPs, which aggravates the degradation of ECM [30]. Herein, similar to data presented by Xu [31], enhanced OS and inflammation were observed in AGEs-stimulated chondrocytes, which were markedly alleviated by Cabozantinib, implying that damage to chondrocytes by AGEs was signally relieved by Cabozantinib. Moreover, ECM components, including type II collagen and aggrecan, in chondrocytes were notably reduced by AGEs, in line with reports by Gu [32]. Following the introduction of Cabozantinib, type II collagen and aggrecan levels were largely increased, implying that the ECM degradation in AGEs-stimulated chondrocytes was ameliorated by Cabozantinib. MMP13 and ADAMTS-5, important enzymes responsible for the degradation of chondrocytes ECM [33, 34], were markedly upregulated in AGEs-stimulated chondrocytes, consistent with Xu’s report [31]. MMP13 and ADAMTS-5 levels were memorably reduced following the introduction of Cabozantinib, implying that Cabozantinib might protect against ECM degradation by suppressing MMP13 and ADAMTS-5 activities.

SOX-9 gene is located on the long arm of human chromosome 17, which is evolutionarily conserved in humans and a variety of animals. SOX-9 participates in embryonic sex determination and is the main transcription factor regulating chondrogenesis during chondrogenic differentiation and development [35]. SOX-9 is a regulatory gene that promotes chondrocyte differentiation and maintains chondrocyte phenotype [36] and has a significant regulatory effect on collagen type II gene [37]. By binding to enhancer sequences of cartilage genes that activate non-cartilage tissue cells, SOX-9 contributes to the development of chondrocyte phenotypes [38]. Herein, SOX-9 was found markedly downregulated in AGEs-stimulated chondrocytes, in line with the data presented by Sun [39]. After the introduction of Cabozantinib, SOX-9 was notably upregulated, implying Cabozantinib might protect against ECM degradation by activating SOX-9. Furthermore, the impact of Cabozantinib on type II collagen and aggrecan levels in AGEs-treated SW1353 chondrocytes was abrogated by silencing SOX-9, indicating that SOX-9 mediated the function of Cabozantinib. In future work, the protective function of Cabozantinib on OA will be further tested using an OA animal model.

Collectively, Cabozantinib prevented AGEs-induced degradation of type 2 collagen and aggrecan in human chondrocytes by mediating SOX-9.

## MATERIALS AND METHODS

### Cells and treatments

SW1353 chondrocytes were obtained from ATCC (USA) and cultured in F-12 medium supplemented with 10% FBS and 1% penicillin-streptomycin antibiotic solution. Cells were cultured in the T75 cell culture flask, the medium was changed 2-3 days. For experiments, cells were used at passages 3-8 and they were stimulated by 100 μg/ml AGEs to establish the *in vitro* OA model. To construct the SOX-9 knockdown SW1353 chondrocytes, cells were transfected with the siRNA targeting SOX-9 (siR-SOX-9) for 48 h, followed by determining the efficacy using the Western blotting assay and then treated with AGEs in presence or absence of Cabozantinib (Cat#T2586, TargetMol, USA).

### 3-(4,5-dimethylthiazol-2-yl)-2,5-diphenyltetrazolium bromide (MTT) assay

Cells in the logarithmic phase were seeded in a 96-well plate and then treated accordingly. 20 μL 5mg/ml MTT (Cat#ab211091, Abcam, UK) was added to each well. After incubation for 4 hours at 37° C, the supernatant was discarded, 150 μL DMSO was loaded, and the crystallization was dissolved after culturing for 2 h. The OD value at 490 nm was determined using a microplate reader (BIOBASE, China).

### 2',7'-dichlorodihydrofluorescein diacetate (DCFH-DA) staining

SW1353 chondrocytes were seeded in a 6-well cell culture plate for 1 day, followed by introducing 200 μL10μM DCFH-DA reagent (Cat#S0033, Beyotime, China) and incubation for half an hour. A microplate reader (BIOBASE, China) was utilized for the detection of the fluorescence value at 488/585 nm.

### The detection of the level of reduced glutathione (GSH)

A commercial kit (Shanghai Yaji Biotechnology Co., LTD, China) was utilized to detect the level of reduced GSH in SW1353 chondrocytes with the sulfhydryl reagent 5,5'-dithio-bis (2-nitrobenzoic acid) (DTNB) method, with the instruction strictly followed.

### ELISA for the detection of interleukin 6 (IL-6), tumour necrosis factor α (TNF-α), type II collagen, aggrecan, MMP-13, and ADAMTS-5 level

Antibodies were loaded onto the well and incubated for 12 h, followed by replacing the reagent with 0.1 mL testing sample or standards. After incubating for 1 h at 37° C, wells were introduced with 0.1 mL of fresh horseradish peroxidase (HRP)-conjugated antibody and cultured for 60 min, followed by adding 0.1 mL temporarily prepared trimethylboroxine (TMB) reagent and cultured for 30 min. After ending the reaction using sulfuric acid, the optical density was checked using the microplate reader (BIOBASE, China) at 450 nm.

### Real-time polymerase chain reaction (PCR)

SW1353 chondrocytes were collected to extract RNAs using Trizol (Cat#16096020, Invitrogen, USA) which were quantified by detecting the absorption at 260 nm. 2 μg RNAs were transformed to cDNA with the PCR reaction (Cat#/RR037A, Takara, Japan). The cDNA was used for quantification utilizing the ABI 7500 Real-time PCR system (Applied Biosystems, USA) using the SYBR green (Sigma, USA). The gene levels were calculated with the 2^-ΔΔCt^ method. GAPDH was used as the housekeeping gene. The following primers were used: SOX-9 forward: 5’-GGAGCTCGAAACTGACTGGAA-3’, SOX-9 reverse: 5’-GGAGCTCGAAACTGACTGGAA-3’; GAPDH forward: 5’-GAGCCCGCA GCCTCCCGCTT-3’, GAPDH reverse: 5’-CCCGCGGCCATCACGCCACAG-3’.

### Western blotting assay

SW1353 chondrocytes were collected to extract proteins, which were quantified by the bicinchoninic acid (BCA) method. After separation using a 12% sodium dodecyl sulfate (SDS)-polyacrylamide gel (PAGE), proteins were moved to the polyvinylidene fluoride (PVDF) membrane, followed by loading with primary antibodies against type II collagen (1:800, Cat#28459-1-AP, Proteintech, USA), Aggrecan (1:1000, Cat#28971, Cell Signaling Technology, USA), SOX-9 (1:800, Cat #82630, Cell Signaling Technology, Boston, USA), or β-actin (1:5000, Cat#sc-69879, SCBT, USA) for 12 hours at 4° C. Then, the secondary antibody (1:2000, SCBT, USA) was introduced and cultured for 90 min, followed by exposure to ECL solution. The Bio-Rad Quantity One software was used for the quantitative analysis of bands.

### Statistical analysis

Data were presented as mean± standard deviation (SD) and were analyzed using a one-way analysis of variance (ANOVA) method. Bonferroni’s method was used as a post-hoc test. P<0.05 was taken as a statistically significant difference.

### Data availability

The data will be made available on reasonable request.
